# Predicting Potential Suitable Habitats of Four *Alisma* Species in China Under Climate Change Scenarios

**DOI:** 10.3390/biology15181649

**Published:** 2026-09-18

**Authors:** Yulu Li, Fu Huang, Qingqing Li, Jinghui Zhang, Shujian Zhang, Chao Peng, Xiaohong Li, Qitao Su

**Affiliations:** Key Laboratory of Jiangxi Province for Biological Invasion and Biosecurity, School of Life Sciences, Jinggangshan University, Ji’an 343009, China

**Keywords:** *Alisma*, MaxEnt model, climate change, potential suitable habitat, medicinal plants

## Abstract

Climate change can alter the potential distribution patterns of species, and the responses of medicinal plants to environmental change are directly relevant to resource conservation and the planning of suitable cultivation regions. Using the MaxEnt model, we combined occurrence records of four *Alisma* species (*Alisma canaliculatum*, *A. gramineum*, *A. orientale*, and *A. plantago-aquatica*) with climatic, topographic, edaphic, and human-activity variables to predict their potential suitable habitats under current conditions and two future periods (2041–2060 and 2081–2100) under three Shared Socioeconomic Pathways (SSP1-2.6, SSP2-4.5, and SSP5-8.5). Two hypotheses were tested: (1) species with narrower thermal niches exhibit greater sensitivity to climate change; and (2) closely related species show significant divergence in dominant environmental factors and spatial responses. All models demonstrated good predictive performance (AUC > 0.9). Dominant environmental factors differed markedly among species: *A. canaliculatum* and *A. orientale* were primarily constrained by temperature-related variables, whereas *A. gramineum* and *A. plantago-aquatica* were more strongly associated with human activity intensity and precipitation-related variables. Consistent with the hypotheses, the potential suitable habitat of *A. orientale* was projected to change most strongly, while that of *A. gramineum* remained the most stable. Under the high-emission scenario, the potential suitable habitat of *A. canaliculatum* was projected to shift northward, accompanied by an increase in total suitable area. *A. orientale* and *A. plantago-aquatica* exhibited internal habitat reorganization, characterized by contraction of low-suitability areas and expansion of high-suitability areas. These findings clarify the niche differentiation patterns of closely related species and identify regions where habitat suitability may be retained, lost, or gained, thereby providing a scientific basis for the adaptive management and conservation planning of these medicinal plants under future climate change.

## Simple Summary

Climate change is altering where plants can grow, and this is especially concerning for medicinal plants. In this study, we used a computer model to predict how the suitable habitats of four water-plantain species (*Alisma*) in China might shift under future climate conditions. We found that each species responds differently. *Alisma gramineum* is expected to remain stable, while *Alisma canaliculatum* may expand northward. *Alisma orientale* and *Alisma plantago-aquatica* may lose some low-quality habitat but gain more high-quality habitat. These results can help guide the conservation and cultivation of these medicinally valuable plants in a changing climate.

## 1. Introduction

The genus *Alisma* (Alismataceae) comprises approximately 11 species worldwide, distributed mainly in temperate and subtropical regions of the Northern Hemisphere, with six species occurring in China [1]. It should be noted that this study follows the taxonomic treatment of Flora of China, in which *Alisma orientale* is recognized as a distinct species. This choice is based on the occurrence data and historical records used in this study, which follow this taxonomic system, and on the fact that the Chinese Pharmacopoeia also lists *A. orientale* as one of the official sources of the traditional medicine “Zexie.” In contrast, POWO treats *A. orientale* as *Alisma plantago-aquatica* subsp.*orientale* (Sam.) Sam. The nomenclature of Flora of China is therefore followed throughout this paper. Most members of this genus are aquatic or palustrine herbs, and several species possess important medicinal, edible, and ecological value [2]. *Alisma canaliculatum* is distributed in the middle and lower reaches of the Yangtze River in China (Figure S1). The whole plant is used medicinally under the traditional Chinese medicine name “Dajian,” with effects of clearing heat, promoting diuresis, detoxifying, and reducing swelling; it is indicated for dysuria, edema, skin blistering, and snake bites [3]. *Alisma gramineum* is a globally widespread species, mainly distributed in north of the Yellow River in China; its tubers possess diuretic, dampness-draining, and heat-clearing properties [1]. *Alisma orientale* is widely distributed in China, mainly produced in Fujian, Sichuan, and Jiangxi. Its dried tuber is one of the official sources of the traditional Chinese medicine “Zexie,” listed as a top-grade drug in the Shennong Bencao Jing. Modern pharmacological studies have demonstrated that the protostane-type triterpenoids (mainly alisols) contained in its tubers exhibit diuretic, anti-inflammatory, hypolipidemic, and antitumor activities. In addition, its scapes can be consumed as a vegetable with certain health benefits [4]. *Alisma plantago-aquatica* is the most widely distributed species of the genus, occurring across Asia, Europe, Africa, North America, and Oceania; its dried tuber is also one of the legal sources of “Zexie,” with diuretic and dampness-draining effects. As an important medicinal economic crop in China, *A. plantago-aquatica* has been widely cultivated in Fujian, Sichuan, and Jiangxi. Its large-scale cultivation not only ensures market supply of medicinal materials but also provides an important pathway for increasing agricultural efficiency and farmers’ income [5]. Therefore, clarifying the key environmental factors limiting the distribution of *Alisma* species and evaluating the suitable habitats of each species under current and future climate scenarios are of considerable practical significance for scientifically planning suitable cultivation regions and rationally guiding introduction and expansion.

Global climate change can alter species’ geographical distribution patterns [6]. Accurately predicting the responses of medicinal plants to future climate is crucial for formulating proactive resource conservation strategies and scientifically planning cultivation zoning [7]. Previous studies on *Alisma* have made progress in phylogeny, chemical constituents, and pharmacological activities [8,9,10]. However, systematic assessments of the potential suitable habitats of *Alisma* species under climate change remain limited. Therefore, this study conducted species-specific predictions and comparative analyses of suitable habitats for four *Alisma* species to accurately characterize their climatic adaptation features.

Species distribution models (SDMs) are important analytical tools in biogeography, conservation biology, and global change ecology, and are widely applied in species distribution prediction, climate change impact assessment, biological invasion risk analysis, and ex situ conservation planning [11]. Among these, the MaxEnt model, based on the maximum entropy principle, has become one of the most widely used species distribution modeling platforms owing to its good compatibility with presence-only data, robust predictive performance, and user-friendly interface [12]. This model estimates the probability distribution of a species by maximizing entropy using species occurrence points and environmental variable data, thereby predicting its potential geographical distribution pattern. In recent years, the MaxEnt model has been widely applied to predict the potential suitable habitats of medicinal plants. For example, Chen et al. predicted the potential and quality distribution of *Anisodus tanguticus* under different climatic conditions on the Qinghai–Tibet Plateau [13], and Hou et al. predicted the potential distribution and climatic response of the endangered medicinal and edible species *Anoectochilus roxburghii* using an optimized MaxEnt model [14]. It should be noted that ecological niche model predictions primarily reflect the responses of potential suitable habitats to changing environmental conditions, rather than being fully equivalent to actual species distribution dynamics.

Among the six species occurring in China, *A. lanceolatum* and *A. nanum* have very few occurrence records, making it difficult to meet the minimum sample size requirements for species distribution modeling. Therefore, this study selected four *Alisma* species with relatively abundant distribution records and distinct geographical distribution patterns—*A. canaliculatum*, *A. gramineum*, *A. orientale*, and *A. plantago-aquatica*—as research objects. The research proposes two hypotheses: (1) species with narrower thermal niches will exhibit greater shifts in potential suitable habitat as climate change intensifies; (2) closely related *Alisma* species will show clear divergence in dominant environmental factors and climatic sensitivity despite partial niche overlap, indicating a degree of niche conservatism rather than complete convergence. Accordingly, this study addresses two levels of research questions: (1) at the fundamental level, we aim to reveal how the environmental factors determining the spatial distributions of the four Alisma species differ, and to what extent their spatial and niche responses diverge under various future climate scenarios; (2) at the applied level, we aim to identify which regions of China are likely to retain, lose, or gain suitability for each species under climate change, and to discuss the implications for conservation and cultivation, thereby providing a theoretical basis for scientifically delineating suitable cultivation areas, rationally adjusting existing cultivation layouts, and mitigating risks. Based on the MaxEnt model and bioclimatic variables under current and future climate scenarios (SSP1-2.6, SSP2-4.5, and SSP5-8.5), we systematically predicted their potential suitable habitat distributions in China and their response trends to future climate change.

## 2. Materials and Methods

**Figure 1:**
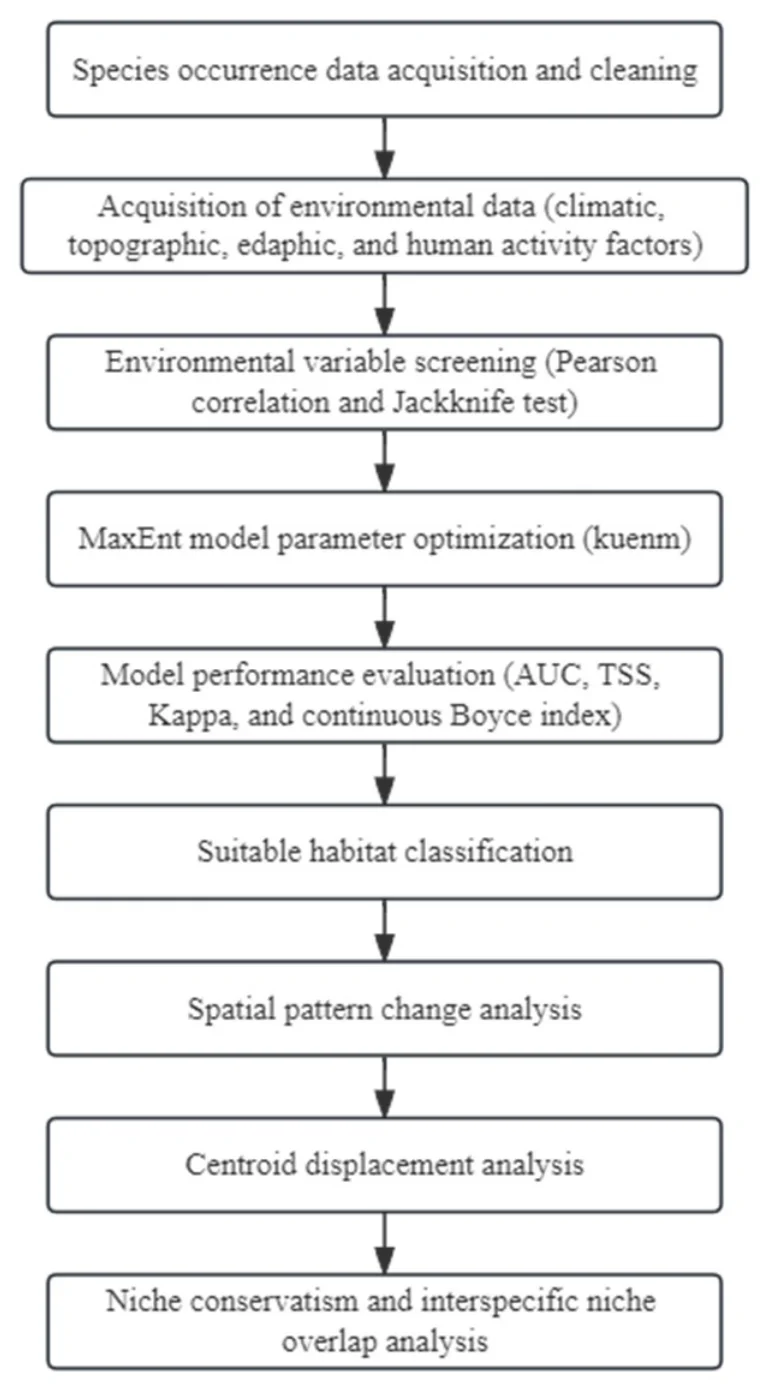
Workflow of this study.

The overall workflow of this study is illustrated in Figure 1, which includes the following main steps: (1) acquisition and cleaning of species occurrence data; (2) acquisition of environmental data, including climatic, topographic, edaphic, and human activity factors; (3) environmental variable screening (Pearson correlation analysis and Jackknife test); (4) MaxEnt model parameter optimization using the kuenm package; (5) model performance evaluation (AUC, TSS, Kappa, and continuous Boyce index); (6) suitable habitat classification; (7) spatial pattern change analysis; (8) centroid displacement analysis; and (9) analysis of niche conservatism and interspecific niche overlap.

### 2.1. Species Occurrence Data

**Figure 2:**
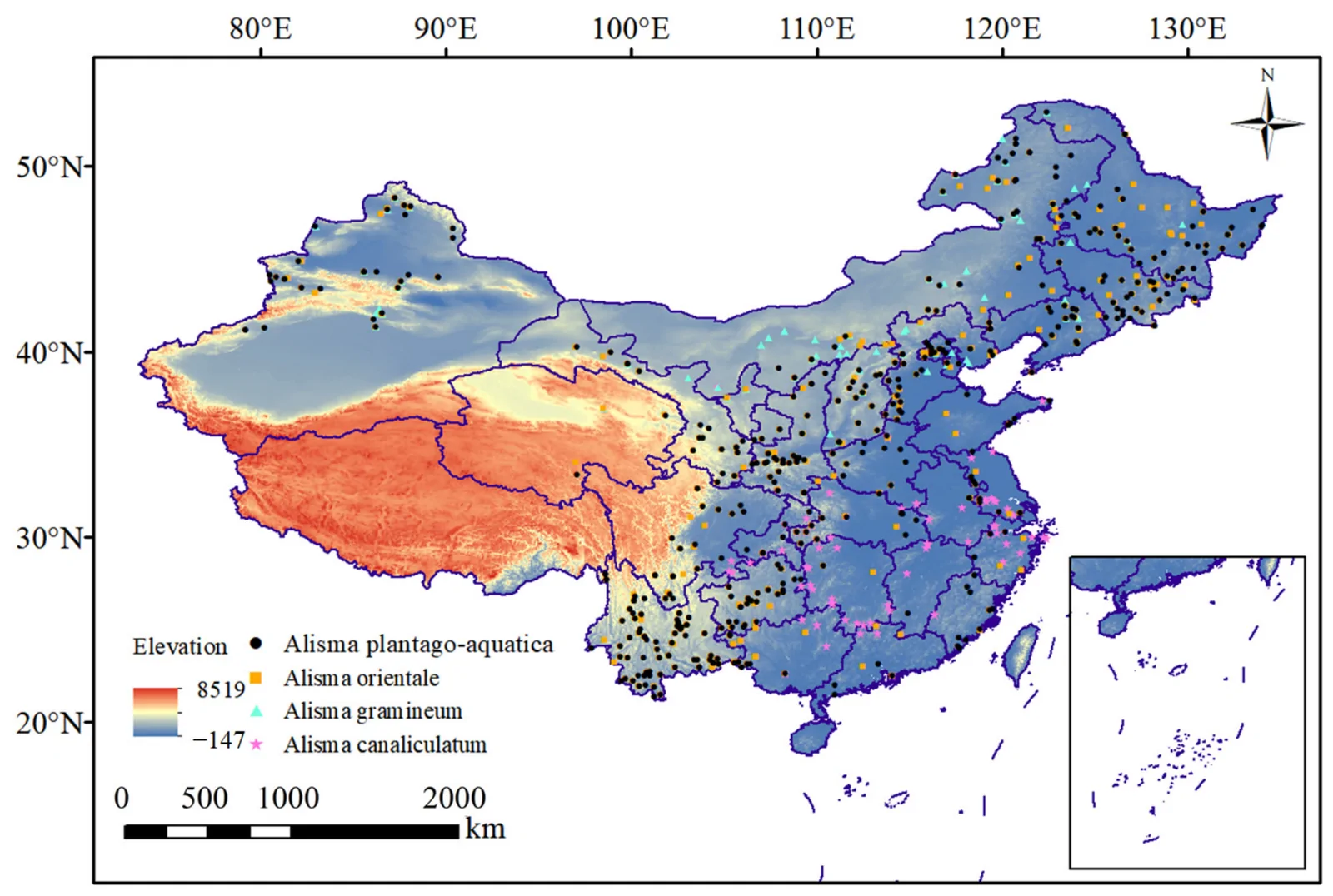
Distribution records of four *Alisma* species (*A. plantago-aquatica*, *A. orientale*, *A. gramineum*, and *A. canaliculatum*) in China. Each point represents one documented occurrence locality after spatial thinning at a 5 km resolution. The background color indicates the elevation gradient, ranging from −147 m to 8519 m.

The four species examined in this study—*A. canaliculatum*, *A. gramineum*, *A. orientale*, and *A. plantago-aquatica*—follow the nomenclature of Flora of China. Occurrence data for *A. canaliculatum*, *A. gramineum*, *A. orientale*, and *A. plantago-aquatica* were obtained through database queries, including the Global Biodiversity Information Facility (GBIF; https://www.gbif.org/, Available online: https://doi.org/10.15468/dl.2cwazv (accessed on 15 January 2026) [15]), the China Digital Herbarium (https://www.cvh.ac.cn/), and the China Plant Image Library (https://ppbc.iplant.cn/), accessed on 7 February 2026. To ensure data quality, only records with precise geographic coordinates were retained, and duplicate records were removed. To reduce sampling bias and spatial autocorrelation, spatial thinning was performed using ENMTools v1.3 (Dan Warren, Rich Glor, and Michael Turelli, available at http://enmtools.com) with a 5 km distance threshold: among points separated by less than 5 km, only one record was retained [16]. After filtering, a total of 91 valid occurrence points were obtained for *A. canaliculatum*, 80 for *A. gramineum*, 337 for *A. orientale*, and 443 for *A. plantago-aquatica* within China (Figure 2). The filtered occurrence records were then organized into .csv files according to species name and coordinates for subsequent MaxEnt model construction and prediction analysis.

### 2.2. Environmental Data Sources and Selection

Environmental variables used for habitat suitability evaluation included climatic, topographic, edaphic, and human disturbance factors (Table S1). Current (1970–2000) and future (2021–2040, 2041–2060, 2061–2080, 2081–2100) climatic data (Bio1–Bio19) were downloaded from the WorldClim database (https://www.worldclim.org/data/index.html, accessed on 12 January 2026) at a spatial resolution of 2.5 arc-minutes [17]. Topographic variables—elevation, aspect, and slope—were derived from a 30 m digital elevation model (DEM) obtained from the Geospatial Data Cloud platform (https://www.gscloud.cn). Soil variables, including soil available water capacity, topsoil pH, soil reference depth, and topsoil texture class, were obtained from the HWSD raster dataset of the Harmonized World Soil Database provided by the Food and Agriculture Organization (http://www.fao.org/soils-portal, accessed on 7 January 2026). Human activity intensity (hf_v2geo1, hereafter referred to as HA) were derived from the Global Human Influence Index, sourced from the Socioeconomic Data and Applications Center (https://catalog.data.gov/dataset/last-of-the-wild-project-version-2-2005-lwp-2-last-of-the-wild-dataset-geographic-8915f, accessed on 9 January 2026). To ensure spatial consistency and compatibility with MaxEnt, all environmental layers were projected to the WGS 1984 coordinate system in ArcGIS 10.8, resampled to a uniform spatial resolution, clipped to the national boundary of China, and converted to ASCII format required for MaxEnt analysis [18]. Future climate projections were based on the BCC-CSM2-MR global climate model under CMIP6. Three Shared Socioeconomic Pathways (SSPs) representing different emission intensities were selected: SSP126 (low-emission scenario), SSP245 (moderate-emission scenario), and SSP585 (very high-emission scenario). Two future periods—2041–2060 and 2081–2100—were selected for analysis [19].

**Figure 3:**
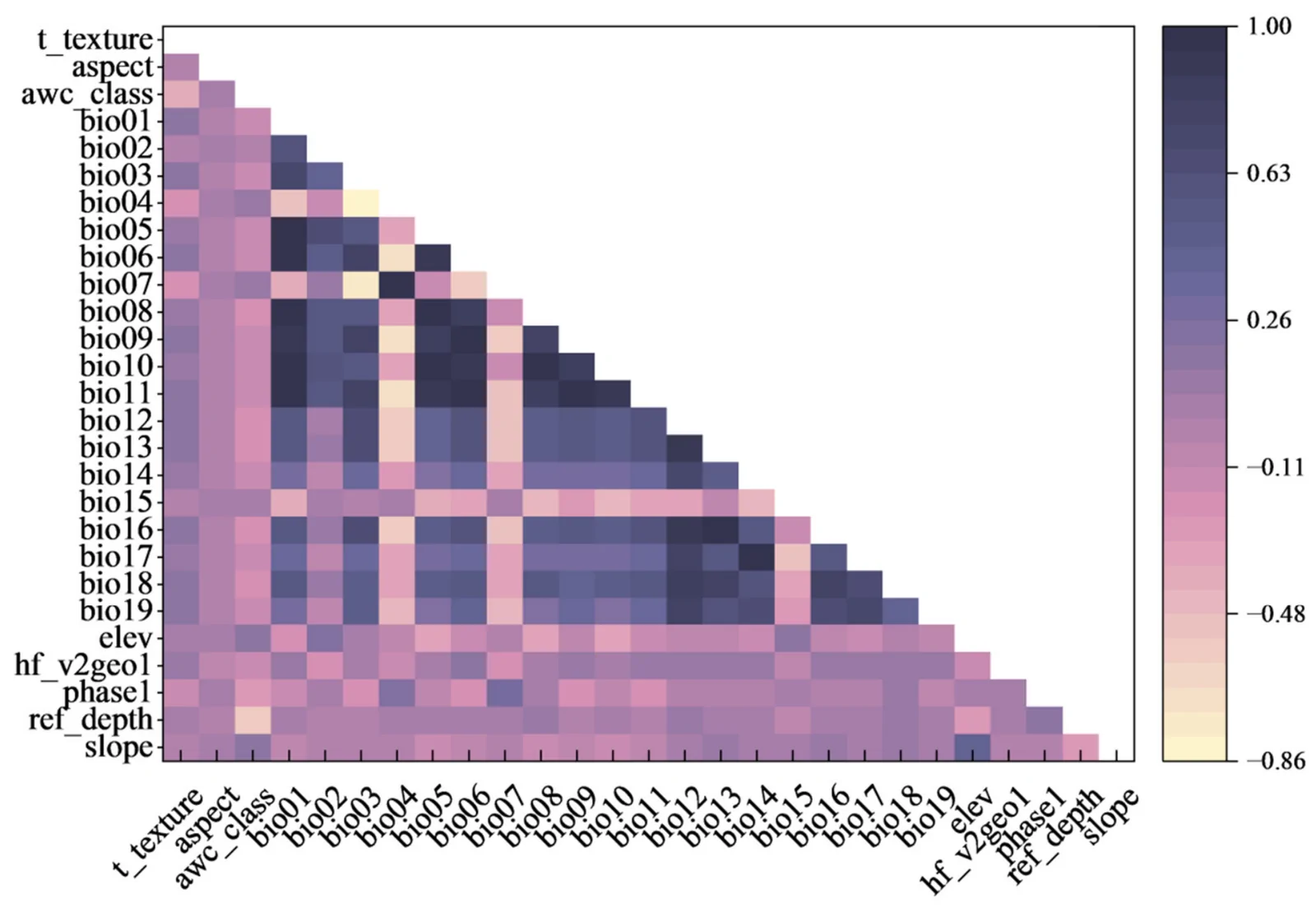
Pearson correlation coefficient heatmap among the 27 environmental variables considered in this study.

To reduce variable collinearity and prevent overfitting, an initial screening of 27 environmental factors was conducted. First, Pearson correlation analysis was used to evaluate correlations among environmental variables, and highly correlated variables (|r| ≥ 0.8) were removed to minimize multicollinearity. The remaining variables were then evaluated using MaxEnt Jackknife analysis to assess their contributions to model predictions. Based on these results and ecological relevance, the final modeling variables were determined [20], including bio03, bio04, bio09, bio11, bio14, bio15, bio18, bio19, aspect, elev, HA, and slope (Figure 3). Full names, abbreviations, and units of these variables are provided in Table S1.

### 2.3. Model Parameter Optimization

To reduce overfitting risk and select the optimal model parameters for each species, MaxEnt model tuning was performed separately for the four *Alisma* species. For each species, candidate models were generated using the kuenm package in R 4.5.1 by systematically varying two key parameters: the regularization multiplier (RM), which ranged from 0.1 to 4, and feature class combinations (FC), which consisted of linear (L), quadratic (Q), product (P), threshold (T), and hinge (H) features [21]. Candidate models were screened using a three-step procedure based on the OR_AICc criterion. First, only models with predictive performance significantly better than random (*p* < 0.05, partial ROC test) were retained. Second, among the models passing the test, those with training omission rates below 10% were selected. Third, the remaining models were ranked by AICc values, and the model with the lowest AICc and ΔAICc < 2 was chosen. If multiple models met these conditions, the one with the fewest parameters was selected following the principle of parsimony [22,23]. Based on the above results, the optimal MaxEnt models for each species were constructed as follows:

(1) The final model for *Alisma canaliculatum* adopted linear and quadratic feature classes (LQ), with a regularization multiplier of 0.7.
(2) The final model for *Alisma gramineum* adopted linear, quadratic, and threshold feature classes (LQT), with a regularization multiplier of 1.3.
(3) The final model for *Alisma orientale* adopted quadratic feature classes (Q), with a regularization multiplier of 0.7.
(4) The final model for *Alisma plantago-aquatica* adopted product, threshold, and hinge feature classes (PTH), with a regularization multiplier of 2.9.

### 2.4. Model Performance Evaluation and Validation

The 12 screened environmental variables and occurrence data were imported into MaxEnt v3.4.1. The jackknife method was used to evaluate the importance of each environmental factor [24]. In each run, 75% of the occurrence records were randomly selected as training data and 25% as test data, and the procedure was repeated 10 times to reduce uncertainty caused by random partitioning. Model predictive performance was evaluated using the receiver operating characteristic (ROC) curve and the area under the curve (AUC). AUC values were used to assess the discrimination ability of the model, with values closer to 1 indicating stronger predictive performance [25].

Model generalization was tested through 5-fold cross-validation: occurrence records were randomly and equally divided into five groups, with four groups used for training and one group retained as an independent test set, iterated five times. At the 10th percentile training presence threshold (TH), three metrics were calculated for each fold: the True Skill Statistic (TSS = Sensitivity + Specificity − 1) [26,27], with the mean across folds reported as a measure of discrimination (TSS > 0.8 is generally considered to indicate good predictive performance); Cohen’s Kappa coefficient (κ = (P_o − P_e)/(1 − P_e)) [26], computed from the confusion matrix and averaged over 10 replicate runs, where higher values indicate stronger agreement between predictions and observations; and the continuous Boyce index, calculated using stratified sampling of the predicted suitability values, 10 moving windows [28,29], and Spearman rank correlation, with the mean across folds reported as a measure of model calibration.

### 2.5. Classification of Suitable Habitats

The ASCII output files for each species were individually imported into ArcGIS, converted to raster format, and used for habitat suitability delineation. To distinguish suitable from unsuitable areas for each species, the SDMToolbox was used to classify potential habitats into three categories based on the 10th percentile training presence threshold (TH): unsuitable (<TH), low-suitability (TH–0.66), and high-suitability (>0.66) [30]. The area and proportion of each category were then calculated. The threshold of 0.66 for high-suitability areas was adopted from widely used classification standards in species distribution modeling studies [31].

### 2.6. Analysis of Spatial Pattern Changes in Suitable Habitats

For each species, the low-suitability and high-suitability areas were merged into a binary map (suitable = 1, unsuitable = 0) based on the habitat suitability classification. In ArcGIS, changes in suitable habitat area, including gains, losses, and spatial distribution, were calculated for each climate scenario. The SDMToolbox was used to track the geometric center displacement of each species’ suitable habitat across time periods [32], providing an overall indication of the spatial response of the distribution to environmental change.

### 2.7. Niche Overlap Analysis

ENMTools v1.3 was used to calculate spatial niche overlap for each species at two levels: (1) niche overlap of the same species between current and future periods, used to assess niche conservatism under climate change; and (2) pairwise niche overlap between different species within the same period, used to assess potential interspecific competition. Niche overlap was quantified using Schoener’s D and Hellinger-based I [33]. Habitat suitability values generated by the MaxEnt model were compared pairwise for each grid cell within the study area using ENMTools. Both metrics range from 0 to 1, with values closer to 1 indicating higher niche overlap.

## 3. Results

### 3.1. Model Accuracy Evaluation

The MaxEnt models for all four *Alisma* species demonstrated good predictive performance (Table 1), adequately reflecting the relationships between environmental variables and the potential distributions of these species. Training AUC values ranged from 0.9192 to 0.9910, and test AUC values ranged from 0.9173 to 0.9878, indicating strong capacity for predicting environmental suitability. In terms of discrimination, the 5-fold cross-validated TSS exceeded the 0.80 threshold for good performance for all species. *Alisma canaliculatum* and *A. gramineum* achieved the highest TSS values, while *A. orientale* and *A. plantago-aquatica* showed slightly lower but still satisfactory discrimination. Regarding classification agreement, the Kappa values for *A. canaliculatum*, *A. gramineum*, and *A. plantago-aquatica* all exceeded 0.80, indicating strong agreement between predictions and observed distributions. The Kappa value for *A. orientale* was 0.6680, representing moderate agreement, which may be related to its relatively narrow niche or smaller number of occurrence points. With respect to model calibration, the continuous Boyce index ranged from 0.2524 to 0.4234 across species, indicating moderate calibration overall. *A. plantago-aquatica* showed the highest calibration, whereas *A. canaliculatum* had the lowest, suggesting acceptable but somewhat uncertain ranking of suitability predictions.

### 3.2. Dominant Environmental Factor Screening and Contribution Rates

The jackknife method was applied to the MaxEnt models, and the results show pronounced interspecific differences among the four *Alisma* species in the percentage contributions of the 12 environmental variables examined. The suitability threshold ranges of the dominant environmental factors for each species are summarized in Table 2.

Pronounced interspecific differences were observed in the dominant environmental factors and their suitable ranges among the four *Alisma* species (Table 2). The distributions of *A. canaliculatum* and *A. orientale* were primarily controlled by temperature-related variables: bio11 contributed most to the former (40.7%), while bio09 contributed most to the latter (36.8%). In contrast, the distributions of *A. gramineum* and *A. plantago-aquatica* were jointly constrained by human activity intensity (hf_v2geo1) and hydrothermal seasonality, with hf_v2geo1 contributing 33.6% and 37.2%, respectively. Precipitation seasonality (bio15) played a role in all four species, but its importance varied considerably. Overall, temperature and moisture conditions jointly defined the niche space of the four species, and human activity intensity exerted a notable influence on the distributions of *A. gramineum* and *A. plantago-aquatica*.

The four *Alisma* species showed significant interspecific divergence in their habitat suitability responses along environmental gradients. The combined response curves consist of multiple panels (Figure S2), each depicting the dynamic changes in habitat suitability of a single species along the gradient of a dominant environmental factor. A predicted habitat suitability probability above 0.5 indicates that the corresponding environmental conditions can support normal growth of the species. *A. canaliculatum* exhibited a typical unimodal response pattern for most climatic factors, with suitability peaking at intermediate environmental ranges and declining rapidly once conditions deviated from the optimum. *A. gramineum* showed a relatively distinct response pattern, with clear threshold effects for some climatic variables (bio19, bio04) and human activity intensity (Table 2), and a comparatively narrow suitable environmental range. *A. orientale* displayed unimodal responses for temperature variables such as bio09 and bio11, but showed continuously increasing suitability along the precipitation seasonality (bio15) gradient, reflecting stronger environmental tolerance. *A. plantago-aquatica* had clear optimal ranges for most environmental factors and exhibited threshold responses to human activity intensity and seasonal climatic factors, with habitat suitability declining substantially once environmental conditions exceeded critical thresholds. Taken together, these four closely related species each had their own environmental optima, but differed markedly in optimal thresholds, tolerance amplitudes, and response curve shapes.

**Figure 4:**
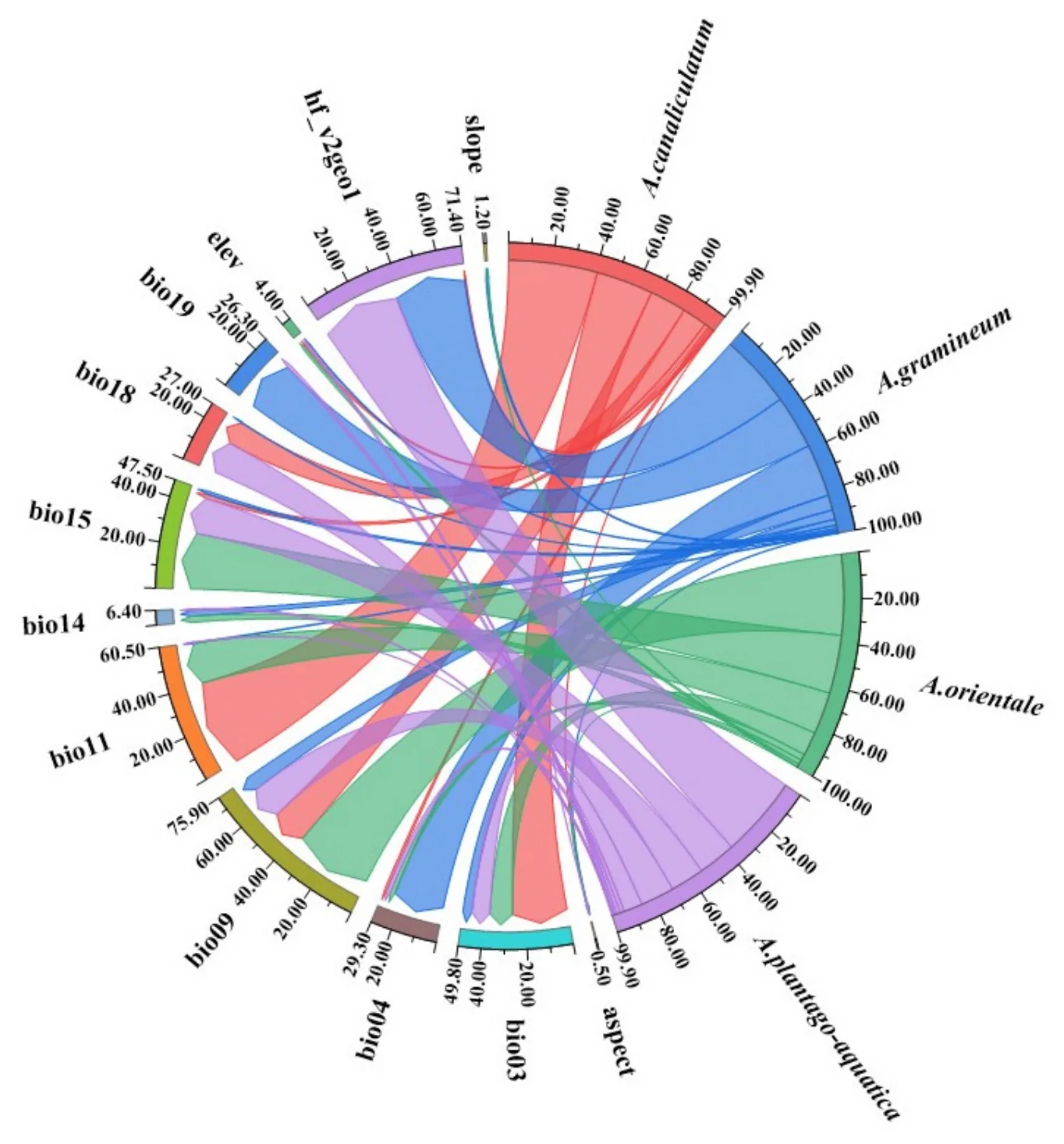
Chord diagram of the contribution rates between the four *Alisma* species and the dominant environmental factors. The outer arc on the left represents the environmental factors that played dominant roles in the MaxEnt models, and the outer arc on the right corresponds to the four *Alisma* species. The connecting bands of different colors represent the associations between environmental variables and particular species. The width of each band indicates the relative contribution of that environmental factor to the species distribution model; wider bands reflect higher contribution rates. Numbers adjacent to the bands represent the percentage contribution values. This figure provides a visual representation of the dominant variable contributions listed in Table 2 and illustrates the niche differences among the *Alisma* species in terms of environmental factor combinations.

Topographic and bioclimatic variables contributed differentially to the geographical distributions of the four *Alisma* species (Figure 4). Bioclimatic variables were the core drivers controlling the broad-scale distribution patterns of all species, whereas topographic factors such as elevation, slope, and aspect, as well as human activity intensity, exhibited strong species-specific effects. *A. canaliculatum* was typically topography- and temperature-dependent. *A. gramineum* was constrained by combined low-temperature and drought stress, reflected in the high contributions of bio19 (24.6%) and bio04 (24.1%). *A. orientale* was regulated by seasonal temperature variation and could tolerate a wider climatic gradient, and *A. plantago-aquatica* responded most strongly to human activity intensity and warm-season precipitation variability. These patterns indicate that clear niche differentiation has occurred among this genus at the regional scale.

### 3.3. Current Potential Suitable Habitats of Alisma Species in China and Their Areas

**Figure 5:**
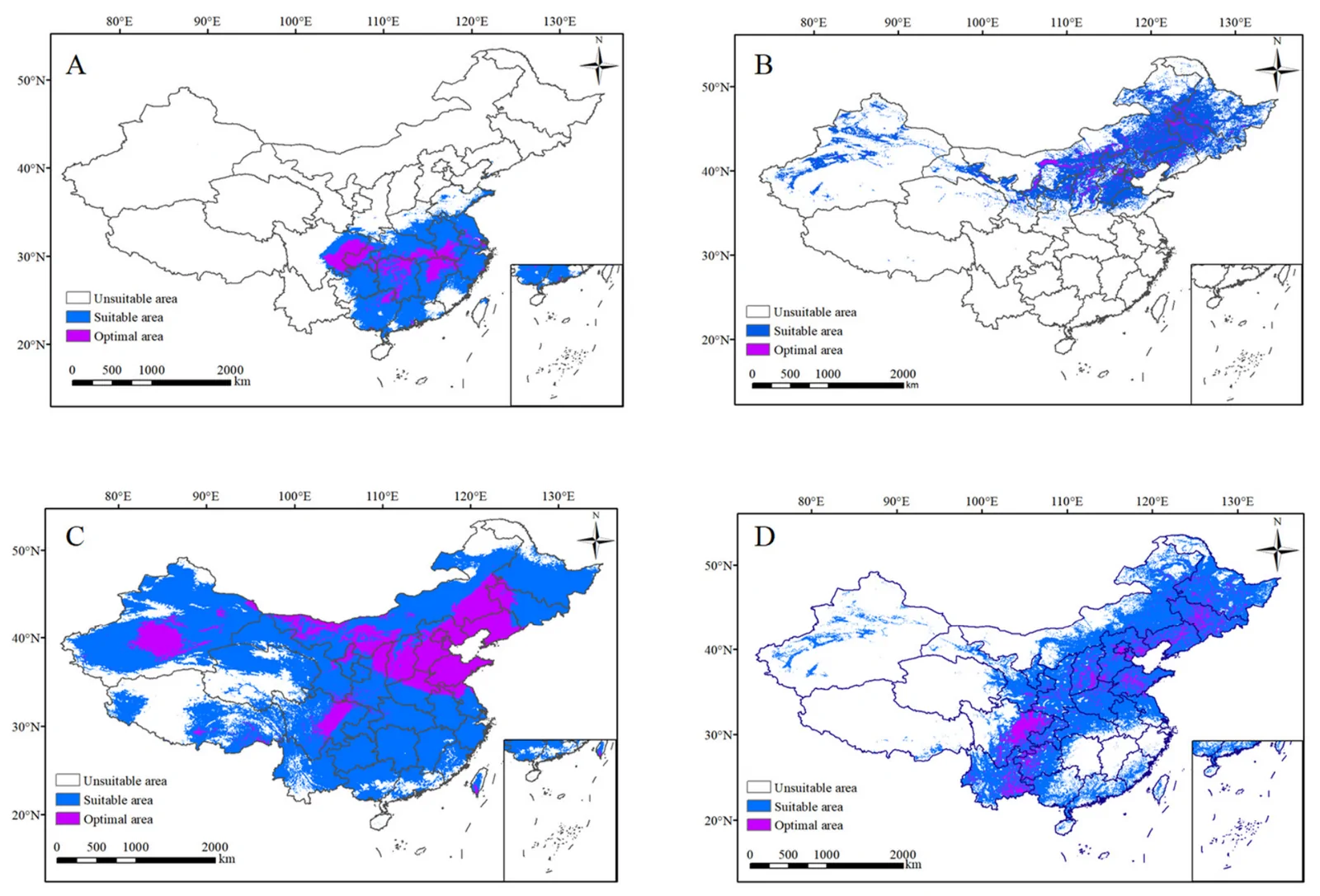
Potential suitable habitats of four *Alisma* species in China under current climatic conditions. The four panels show the potential suitable distribution ranges of *Alisma canaliculatum* (**A**), *A. gramineum* (**B**), *A. orientale* (**C**), and *A. plantago-aquatica* (**D**). In the legend, white areas represent unsuitable area, blue areas represent suitable area, and purple areas represent optimal area.

Under current climatic conditions, the potential suitable habitats of the four *Alisma* species in China are shown in Figure 5. The potential suitable habitat of *Alisma canaliculatum* was concentrated in the southern subtropical region, with high-suitability areas mainly located in the Sichuan Basin, the middle reaches of the Yangtze River, and northern South China; most of northern China was unsuitable. The potential suitable habitat of *A. gramineum* extended in a zonal pattern from the northwest to the northeast, with high-suitability areas concentrated in North China, eastern Inner Mongolia, and the Northeast Plain, and only scattered suitable patches in the south. *A. orientale* had the broadest potential suitable habitat among the four species, spanning the eastern region from south to north; its high-suitability areas were concentrated in the North China Plain and the eastern Loess Plateau, with scattered occurrences in the southwest. The potential suitable habitat of *A. plantago-aquatica* broadly covered the eastern monsoon region, with high-suitability areas mainly distributed in the Sichuan Basin, the eastern Yunnan–Guizhou Plateau, and the upper reaches of the Yangtze River. Under current climatic conditions, the total potential suitable areas of each species were 177.305 × 10^4^ km^2^ for *A. canaliculatum*, 178.835 × 10^4^ km^2^ for *A. gramineum*, 754.749 × 10^4^ km^2^ for *A. orientale*, and 394.698 × 10^4^ km^2^ for *A. plantago-aquatica*.

### 3.4. Future Changes in Potential Suitable Habitats of Alisma Species in China

**Figure 6:**
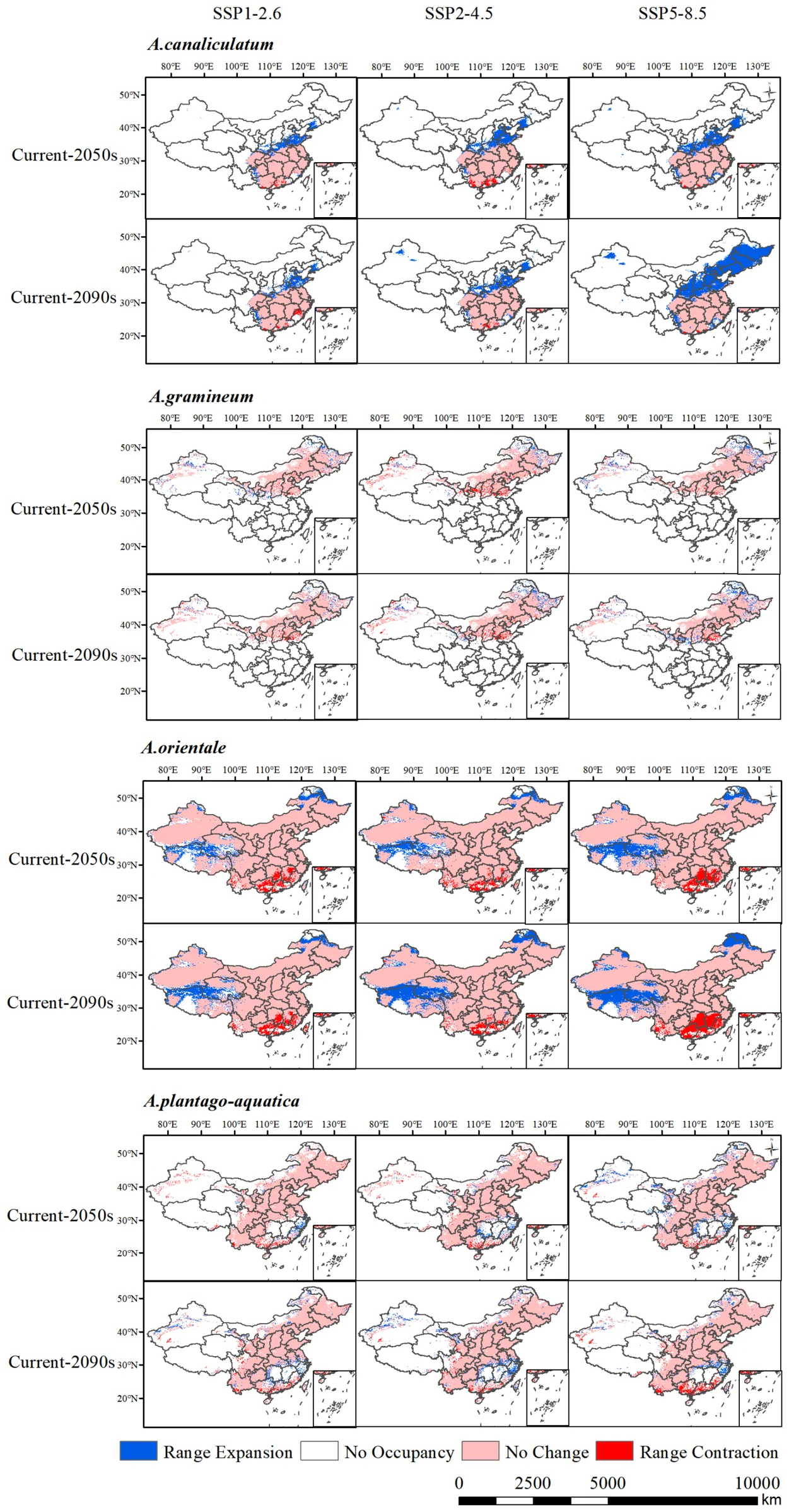
Spatial dynamics of potential suitable habitats of four *Alisma* species under future climate scenarios. The four panels show changes in potential suitable habitat of *Alisma canaliculatum*, *A. gramineum*, *A. orientale*, and *A. plantago-aquatica* between the current period and 2041–2060 and between the current period and 2081–2100. For each species, three columns correspond to three future climate scenarios (SSP1-2.6, SSP2-4.5, and SSP5-8.5). Blue (Range Contraction) indicates expansion of potential suitable habitat; white (No Occupancy) indicates no presence; pink (No Change) indicates stable suitable areas; and red (Range Contraction) indicates habitat contraction.

Compared with current climatic conditions, all four species exhibited divergent spatial shifts in suitable habitats under the three emission scenarios during 2041–2060 (2050s) (Figure 6, Table 3).

The current potential suitable habitat of *A. canaliculatum* is concentrated in southern China. During 2041–2060, the total suitable habitat expansion area increased continuously with increasing emission intensity. By 2081–2100, the expansion area under the high-emission SSP5-8.5 scenario was far greater than under the other two scenarios. The low-suitability area increased to 161.965 × 10^4^ km^2^, and the high-suitability area increased substantially to 193.800 × 10^4^ km^2^, resulting in a total suitable area of 355.765 × 10^4^ km^2^, which was significantly higher than the current value (177.305 × 10^4^ km^2^). Across all scenarios, the total suitable habitat contraction area for this species remained at a very low level, with only small-scale contraction occurring locally in the southern portion of its native range.

The current potential suitable habitat of *A. gramineum* is concentrated in northern and northeastern China. In both future periods and under all three climate scenarios, its expansion area was far smaller than those of the other three species, with only limited expansion occurring along the northern margins of its existing distribution. With increasing emission intensity, the expansion area increased slightly under the long-term SSP5-8.5 scenario, while contracted patches were scattered within the original distribution. Overall, the total suitable habitat area of *A. gramineum* fluctuated only within a narrow range under future climate scenarios, indicating the most stable distribution pattern among the four species, with no evidence of large-scale expansion or decline.

*A. orientale* had the broadest current potential suitable habitat among the four species, widely occupying eastern, southern, and southwestern China. During 2041–2060, the total suitable habitat expansion area increased with emission intensity. By 2081–2100, the expansion areas under SSP2-4.5 and SSP5-8.5 increased further and significantly. Under the long-term SSP5-8.5 scenario, the low-suitability area decreased from the current 597.284 × 10^4^ km^2^ to 510.820 × 10^4^ km^2^, while the high-suitability area increased sharply from 157.465 × 10^4^ km^2^ to 303.523 × 10^4^ km^2^. Consequently, the total suitable area increased from 754.749 × 10^4^ km^2^ to 814.343 × 10^4^ km^2^. However, the contracted area of this species reached the highest value among all scenarios (72.083 × 10^4^ km^2^), with extensive contraction of high-suitability areas in southern China, reflecting a spatial reorganization characterized by low-suitability contraction and high-suitability expansion.

The current potential suitable habitat of *A. plantago-aquatica* is concentrated in central, southwestern, and southern North China. During 2041–2060, modest northward expansion occurred under all three scenarios; during 2081–2100, expansion further increased under SSP2-4.5. Under the long-term high-emission SSP5-8.5 scenario, the low-suitability area decreased to 279.038 × 10^4^ km^2^, while the high-suitability area increased to 106.716 × 10^4^ km^2^. The total suitable area decreased slightly from the current 394.698 × 10^4^ km^2^ to 385.754 × 10^4^ km^2^, with a marked increase in contracted area and obvious contraction of low-suitability areas in southern South China. Nevertheless, the high-suitability area increased significantly, indicating improved core habitat quality.

Comparing the two future periods, the long-term high-emission SSP5-8.5 scenario drove the most pronounced divergence in species distribution patterns. The potential suitable habitat of *A. canaliculatum* was projected to shift northward, the potential suitable habitat of *A. gramineum* remained stable, and the potential suitable habitats of *A. orientale* and *A. plantago-aquatica* exhibited a latitudinal shift characterized by northern margin expansion and southern native habitat contraction. Under the low-emission SSP1-2.6 scenario, habitat changes remained generally moderate for all four species. With increasing greenhouse gas emissions, spatial differences in species distributional responses intensified.

### 3.5. Centroid Displacement

**Figure 7:**
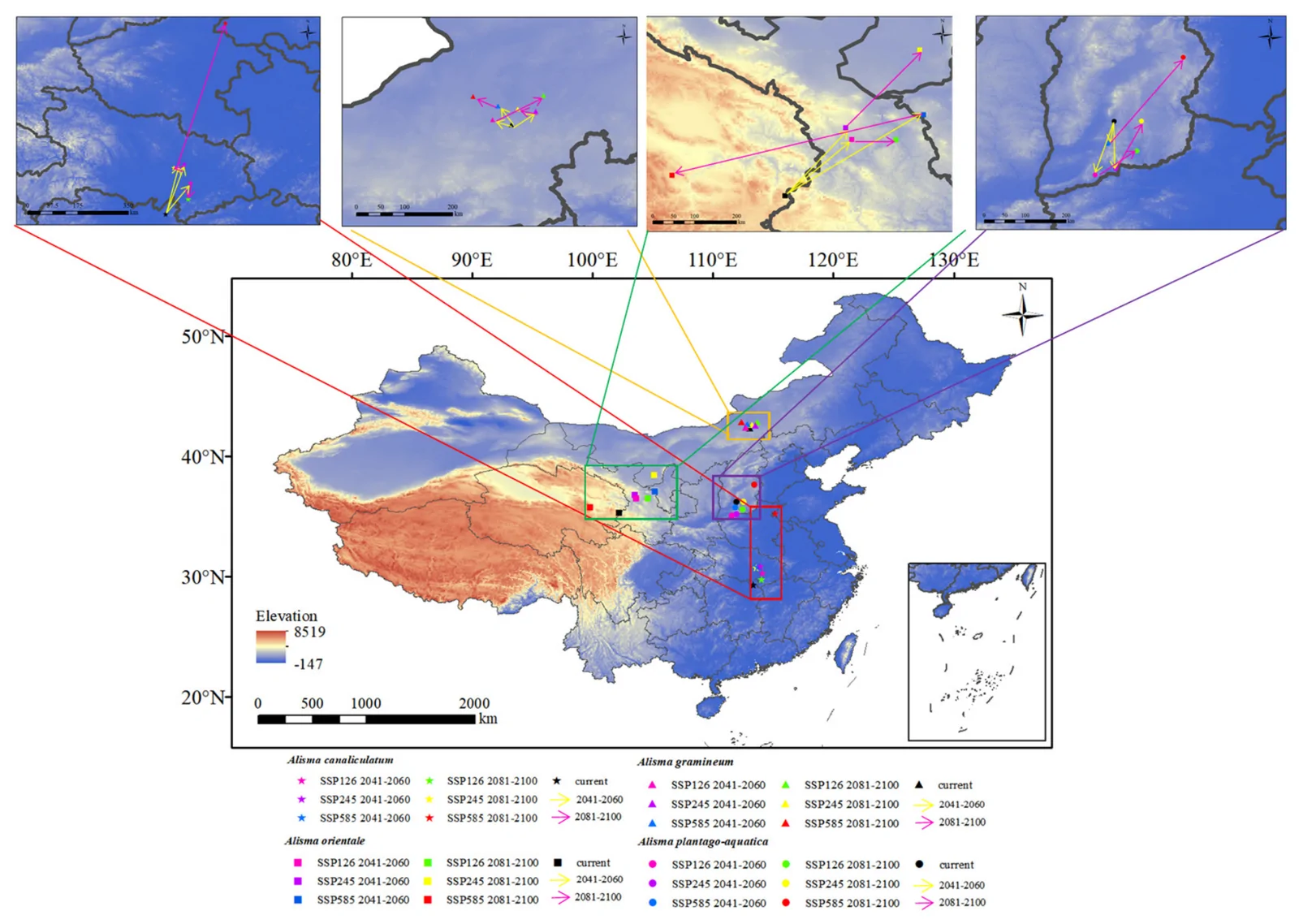
Geographic displacement trajectories of the distribution centroids of four *Alisma* species under future climate scenarios. The main map uses an elevation background (from −147 m to 8519 m), with a red-to-blue gradient indicating high to low elevation. The four colored rectangles (red, green, yellow, and purple) correspond to the four enlarged inset panels with matching colored borders, showing details of representative areas in China. The legend indicates the centroid positions of the four species (*A. canaliculatum*, *A. gramineum*, *A. orientale*, and *A. plantago-aquatica*) under different climate pathways (SSP1-2.6, SSP2-4.5, and SSP5-8.5) and future periods (2041–2060 and 2081–2100), along with the displacement directions relative to their current distributions.

Based on the MaxEnt model predictions, this study tracked the geometric center displacement of the suitable distribution areas of the four *Alisma* species under different climate scenarios (Figure 7). Under current conditions, the centroid coordinates of each species were as follows: *A. canaliculatum*, 113.322° E, 29.299° N; *A. gramineum*, 113.085° E, 42.286° N; *A. orientale*, 102.230° E, 35.305° N; and *A. plantago-aquatica*, 111.967° E, 36.228° N. The displacement distances and directions between time periods are described below.

From the current period to the 2050s, the centroid of *A. canaliculatum* shifted consistently northeastward under all three emission scenarios: under SSP1-2.6, it moved 130.292 km to 114.084° E, 30.270° N; under SSP2-4.5, it moved 180.929 km to 113.897° E, 30.853° N; and under SSP5-8.5, it moved 170.453 km to 113.679° E, 30.805° N. From the 2050s to the 2090s, the centroid retreated southwestward by 56.768 km under SSP1-2.6 to 114.022° E, 29.760° N, moved southwestward by 32.748 km under SSP2-4.5 to 113.578° E, 30.746° N, and continued to shift dramatically northeastward by 513.583 km under SSP5-8.5 to 115.181° E, 35.260° N, with latitude migrating from 29.299° N to 35.260° N, reflecting a pronounced northward shift in potential suitable habitat under strong warming.

For *A. gramineum*, centroid displacement from the current period to the 2050s was extremely limited: under SSP1-2.6, it moved northwestward by 30.714 km to 112.733° E, 42.377° N; under SSP2-4.5, it moved northeastward by 46.337 km to 113.537° E, 42.535° N; and under SSP5-8.5, it moved northwestward by 43.785 km to 112.833° E, 42.634° N. From the 2050s to the 2090s, the centroid moved northeastward by 92.971 km under SSP1-2.6 to 113.687° E, 42.829° N, northwestward by 27.993 km under SSP2-4.5 to 113.201° E, 42.576° N, and northwestward by 42.687 km under SSP5-8.5 to 112.370° E, 42.810° N. All displacement distances for this species remained below 100 km, indicating an exceptionally stable pattern of potential suitable habitat.

For *A. orientale*, from the current period to the 2050s, the centroid shifted northeastward across all three emission scenarios, with displacement distance increasing markedly with emission intensity: under SSP1-2.6, it moved 186.073 km to 103.661° E, 36.512° N; under SSP2-4.5, it moved 199.507 km to 103.529° E, 36.761° N; and under SSP5-8.5, it moved 328.801 km to 105.193° E, 37.040° N. From the 2050s to the 2090s, the centroid moved southeastward by 84.758 km under SSP1-2.6 to 104.607° E, 36.507° N, continued northeastward by 231.909 km under SSP2-4.5 to 105.118° E, 38.425° N, and sharply reversed direction under SSP5-8.5, retreating southwestward by 503.576 km to 99.811° E, 35.748° N. This dramatic retreat was consistent with the extensive contraction of core high-suitability habitat in southern China observed under this scenario.

For *A. plantago-aquatica*, centroid displacement from the current period to the 2050s exhibited directional divergence: under SSP1-2.6, it moved southwestward by 135.795 km to 111.550° E, 35.052° N; under SSP2-4.5, it moved southeastward by 115.404 km to 112.000° E, 35.188° N; and under SSP5-8.5, it moved southwestward by 55.347 km to 111.831° E, 35.741° N. From the 2050s to the 2090s, the centroid shifted consistently northeastward under all three scenarios: by 101.625 km under SSP1-2.6 to 112.466° E, 35.576° N; by 126.604 km under SSP2-4.5 to 112.565° E, 36.232° N; and by 256.595 km under SSP5-8.5 to 113.484° E, 37.632° N. This “southward retreat followed by northward advance” pattern may reflect a phased response of this species to climate change.

Collectively, among the four *Alisma* species, the potential suitable habitat centroid of *A. gramineum* was the most stable and was least affected by climate change; *A. canaliculatum* and *A. orientale* displayed substantial displacement under high-emission scenarios, with their core distribution areas facing significant spatial reorganization; and *A. plantago-aquatica* exhibited a phased fluctuation pattern. These results indicate that the driving forces of climate change on the distribution patterns of different *Alisma* species are fundamentally distinct, and that closely related species may have undergone marked divergence in their spatial response strategies.

### 3.6. Niche Conservatism and Interspecific Niche Differentiation

**Figure 8:**
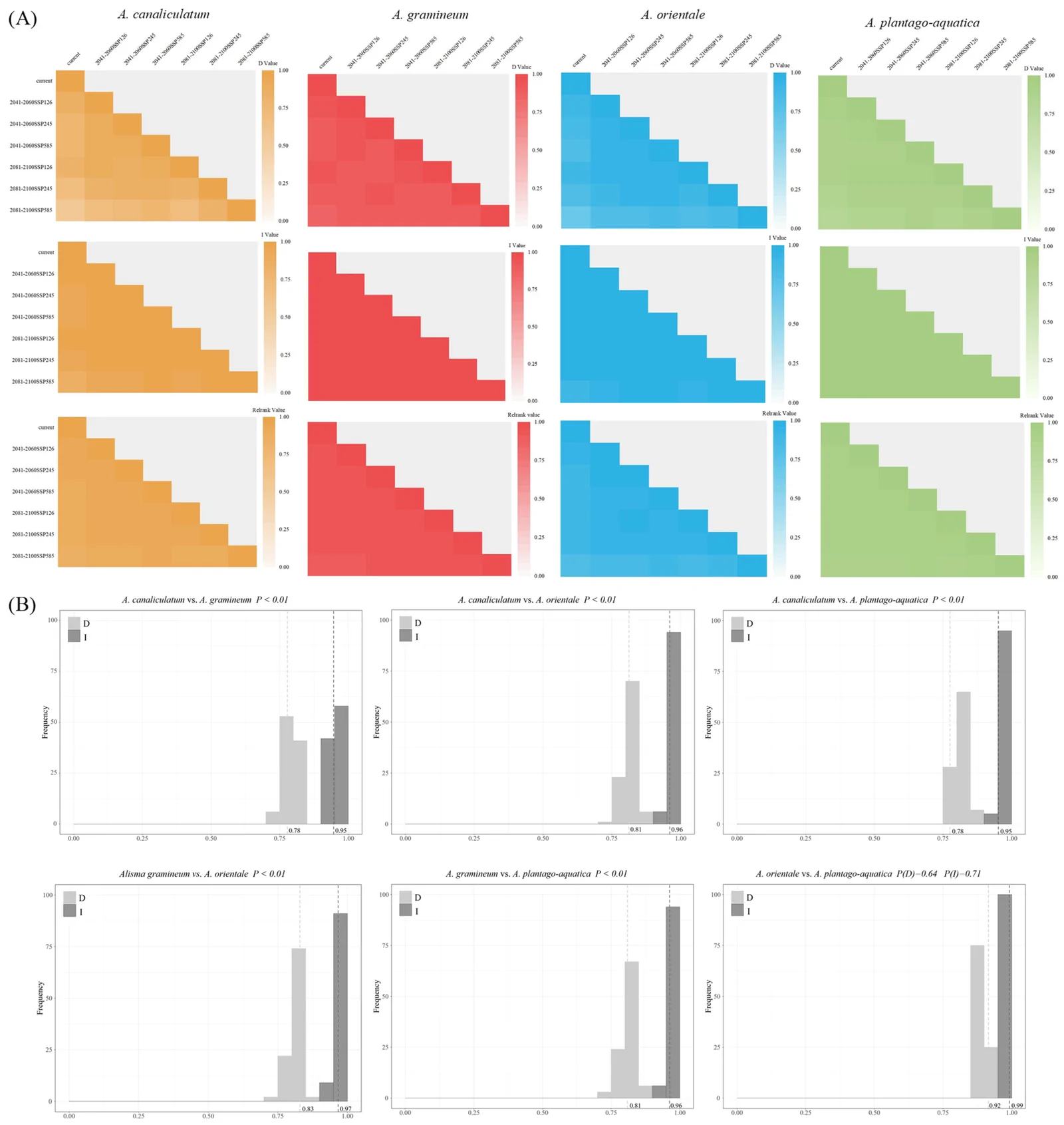
Niche similarity and interspecific niche equivalency tests among the four *Alisma* species under different climate scenarios. (**A**) Pairwise comparison heatmap of niche similarity. The 12 heatmaps are arranged in four columns from left to right, corresponding to the four *Alisma* species (*A. canaliculatum*, *A. gramineum*, *A. orientale*, and *A. plantago-aquatica)*. Three rows from top to bottom represent Schoener’s D, Hellinger-based I (Warren’s I), and relative rank (Relrank), respectively. In each heatmap, the x- and y-axes include combinations of the species across three time periods (current, 2041–2060, and 2081–2100) and three future climate scenarios (SSP1-2.6, SSP2-4.5, and SSP5-8.5). Each cell indicates the niche overlap value between two species under a specific climate scenario, with colors approaching red indicating higher similarity (scale: 0.00–1.00). (**B**) Frequency distributions and equivalence tests of pairwise niche overlap indices (Schoener’s D and Hellinger-based I, the latter also known as Warren’s I). Six panels correspond to all six pairwise combinations of the four species. Light gray and dark gray histograms represent the frequency distributions of the D and I indices, respectively, across replicate model runs. The *p* value in each panel indicates the result of the niche equivalency test. Dashed lines indicate the observed values of D and I.

Based on the background similarity test and niche identity test implemented in ENMTools, the niche relationships of the four *Alisma* species were evaluated from two dimensions: (1) niche conservatism of the same species across different climate scenarios (Schoener’s D and Hellinger’s I), reflecting the longitudinal impact of climate change on species’ niche space (Figure 8); and (2) interspecific niche overlap among different species under current climatic conditions, revealing the horizontal niche relationships among species (Figure 8).

With respect to niche conservatism, the four species exhibited a clear gradient. *Alisma gramineum* showed the strongest conservatism, with D values consistently above 0.86 and I values above 0.97 between the current period and all future scenarios, indicating that climate change had very limited effects on its niche space. *Alisma canaliculatum* ranked second, with D values above 0.83 (I > 0.94) between the current period and all three 2041–2060 scenarios, indicating well-maintained niche integrity; however, under SSP5-8.5 during 2081–2100, D decreased to 0.582 and I to 0.866, suggesting that a substantial niche shift may occur under long-term high-emission conditions. *Alisma plantago-aquatica* showed intermediate niche conservatism, with D = 0.815 and I = 0.963 under SSP5-8.5 during 2081–2100; although high similarity was maintained, a discernible declining trend relative to 2041–2060 was evident. *Alisma orientale* exhibited the most pronounced niche shift, with both D and I values decreasing systematically with increasing emission intensity: D declined from 0.830 under 2041–2060 SSP5-8.5 to 0.693 under 2081–2100 SSP5-8.5, and I decreased from 0.971 to 0.908—the largest decline among the four species—indicating the highest risk of niche reorganization under high-emission scenarios.

With regard to interspecific niche relationships, the niche identity test revealed divergent patterns among the six species pairs. The observed D values of five pairs—*A. canaliculatum* vs. *A. gramineum*, *A. canaliculatum* vs. *A. orientale*, *A. canaliculatum* vs. *A. plantago-aquatica*, *A. gramineum* vs. *A. orientale*, and *A. gramineum* vs. *A. plantago-aquatica*—were concentrated in the 0.90–1.00 range, significantly exceeding the null model expectation (0.75–0.85), indicating high niche overlap beyond background environmental similarity. In contrast, the *A. orientale*–*A. plantago-aquatica* pair did not reach statistical significance in the niche identity test (P(D) = 0.64, P(I) = 0.71), and the null hypothesis of niche identity could not be rejected, suggesting that the niche overlap between these two species could not be statistically distinguished from random expectation. Although numerically all species pairs showed relatively high overlap, statistically the niche similarity between *A. orientale* and *A. plantago-aquatica* is more likely driven by shared environmental backgrounds rather than representing a significant signal of niche conservatism or convergence. It should also be noted that the niche identity test is based solely on abiotic environmental space; actual differentiation among species in microhabitat, phenology, or resource use dimensions requires further investigation.

## 4. Discussion

This study systematically evaluated changes in the potential suitable habitats of four *Alisma* species in China under current and future climate scenarios using the MaxEnt model. Two hypotheses were tested: (1) species with narrower thermal niches are more sensitive to climate change and will experience greater shifts in potential suitable habitat; and (2) closely related species show significant divergence in dominant environmental factors and climatic sensitivity despite partial niche overlap. The results supported both hypotheses. The distributions of the four species were differentially regulated by distinct environmental factors, reflecting adaptive differentiation in niche dimensions within the genus. Temperature-related variables were the primary factors influencing the predicted potential distributions of *A. canaliculatum* and *A. orientale*, which is consistent with the general sensitivity of subtropical wetland plants to low temperatures [34]. In contrast, human activity intensity contributed markedly to the models for *A. gramineum* and *A. plantago-aquatica* (33.6% and 37.2%, respectively), suggesting that human disturbance may substantially affect their potential distributions. Human activities can reshape the suitable habitats of *Alisma* species by altering wetland hydrological connectivity, increasing water eutrophication [35], and directly damaging riparian habitats. This finding aligns with recent studies emphasizing the importance of human activity variables in species distribution models [36], and indicates that for plant taxa dependent on wetland and aquatic habitats, human activity intensity has become a spatial constraint of comparable importance to climatic factors. Notably, soil factors were not identified as dominant limiting factors for any of the four species, which may reflect the general adaptation of *Alisma* species to wetland habitats: at broader spatial scales, water availability and seasonal thermal regimes, rather than soil type, determine their distribution boundaries.

The niche identity test indicated that all six species pairs showed high niche overlap in abiotic environmental space (Schoener’s D values concentrated in the range of 0.90–1.00), reflecting the shared preference of closely related species for similar wetland habitats [37]. Among them, the niche identity test for *A. orientale* and *A. plantago-aquatica* did not reach statistical significance (*p* > 0.05); the possibility that their niches are identical cannot be excluded, suggesting that the environmental requirements of these two species are highly similar in the current climatic space and that they may have a strong competitive relationship [38]. However, because this study was based solely on abiotic factors such as climate, topography, and human activities, the actual degree of niche differentiation between these two species in microhabitat selection, phenological differentiation, or resource use dimensions still requires verification through population surveys and ecological experiments in their sympatric distribution areas.

Under future climate scenarios, the four species exhibited markedly divergent changes in suitable habitat. The potential suitable habitat of *A. gramineum* was projected to remain highly stable, with centroid displacement not exceeding 100 km across all scenarios and minimal fluctuations in suitable habitat area. This stability may be related to its northern distribution preference: this species mainly occurs in North China and Northeast China, where although warming amplitudes are large under future climate change, moisture conditions may remain within its tolerance range. The potential suitable habitat of *A. canaliculatum* was projected to shift northward under the SSP5-8.5 high-emission scenario, with its centroid shifting substantially northeastward by 513.58 km and both suitable and optimal areas increasing significantly. This expansion pattern may benefit from climate warming releasing previously low-temperature-limited high-latitude habitats [39,40].

Climate change has already caused significant shifts in species distribution patterns worldwide [41], and among the four species examined here, the potential suitable habitat of *A. orientale* was projected to change most strongly. Under SSP5-8.5, its low-suitability area decreased from the current 597.28 × 10^4^ km^2^ to 510.82 × 10^4^ km^2^, while its high-suitability area increased sharply from 157.47 × 10^4^ km^2^ to 303.52 × 10^4^ km^2^. This change reflects an internal quality reorganization of suitable habitat—loss of low-suitability areas accompanied by a significant increase in high-suitability areas [42]. Such “low-suitability contraction, high-suitability expansion” spatial reorganization may reflect the dual effects of climate warming [43]: on the one hand, low-suitability areas at the southern margins of the original distribution are lost because heat exceeds the species’ tolerance limits; on the other hand, optimized hydrothermal combinations in the core distribution area upgrade some low-suitability areas to high-suitability areas, while new habitats at the northern margins are released [44]. This “quality-for-quantity” pattern is consistent with the internal reorganization mode of suitable habitats reported for widespread species under climate change. The potential suitable habitat of *A. plantago-aquatica* showed a similar divergent trend, with total suitable area contracting while high-suitability area increased. Overall, climate change is driving the spatial redistribution of biodiversity [27].

Centroid analysis quantified the overall migration trend of suitable habitat from a spatial perspective. Centroid displacement is not a direct simulation of actual population migration but rather a landscape-level signal reflecting the spatial tendency of the climatic envelope. The highly stable centroid of *A. gramineum* further confirms its distributional conservatism. The substantial displacements of *A. canaliculatum* and *A. orientale* under the high-emission scenario indicate that their core distribution areas face significant spatial reorganization risks. The phased “southward retreat followed by northward advance” pattern of *A. plantago-aquatica* may reflect differential responses to dominant climatic factors across time periods: recent changes in precipitation patterns may have temporarily compressed the southern suitable areas, while rising temperatures in the long term drive the overall northward migration of suitable habitats [45]. However, the specific mechanisms require further verification.

Niche conservatism between current and future climate scenarios exhibited gradient differentiation consistent with spatial response patterns. The Schoener’s D values of *A. gramineum* exceeded 0.86 across all scenarios, indicating the most conservative niche. The D values of *A. orientale* decreased systematically with increasing emission intensity, dropping to 0.693 under the SSP5-8.5 scenario during 2081–2100, the lowest among all four species. This trend, together with its broadest current distribution range and largest centroid displacement, suggests that widespread species may not necessarily have the most stable niches under climate change—on the contrary, their broad distribution may depend precisely on specific climatic envelopes, and when climatic conditions exceed their tolerance thresholds, suitable habitat may undergo dramatic reorganization [46].

Several limitations of this study should be acknowledged. First, the occurrence datasets are relatively limited, which may constrain the models’ ability to fully characterize the environmental tolerance of each species. Second, future climate projections were based on a single global climate model, which cannot capture inter-model uncertainty. Third, as a correlative approach, MaxEnt does not account for dispersal capacity, biotic interactions, or adaptive evolution; the projected suitable areas should therefore be interpreted as potential habitat rather than realized distribution. Future work should prioritize expanding occurrence databases, employing multi-model ensemble climate projections, and integrating field surveys with population dynamic monitoring to further validate and refine model predictions. In addition, the continuous Boyce index values of all species in this study were generally low (0.05–0.42), reaching only a moderate calibration level. This may be related to the fact that *Alisma* species are widespread wetland plants whose suitable habitats often occur as discrete small patches, limiting the consistency between predicted suitability rankings and independent validation data. Meanwhile, occurrence point biases and the resolution of environmental variables (especially soil and human activity data) may also introduce uncertainty. Future work should improve model calibration through higher-resolution hydrological and land-use data.

## 5. Conclusions

This study revealed the potential suitable habitat patterns and interspecific differentiation of four *Alisma* species in China under current and future climate scenarios. At the fundamental level, the results clarify how closely related species diverge in environmental drivers and climatic sensitivity, supporting the two proposed hypotheses. At the applied level, the projections identified regions where habitat suitability may be retained, lost, or gained. The main conclusions are as follows: (1) The four species were regulated by distinct environmental factors. *A. canaliculatum* and *A. orientale* were mainly constrained by temperature variables, whereas *A. gramineum* and *A. plantago-aquatica* were jointly influenced by human activity intensity and climatic factors. (2) Current suitable habitats showed clear spatial differentiation along a south–north gradient. *A. canaliculatum* was concentrated in the southern subtropical region; *A. gramineum* was mainly distributed in northern and northeastern China; *A. orientale* had the broadest suitable habitat, covering most of eastern, central, and southern China; and *A. plantago-aquatica* was concentrated mainly in the eastern monsoon region. (3) Under future climate scenarios, the potential suitable habitat changes showed a clear gradient of climate sensitivity. The potential suitable habitat of *A. gramineum* remained the most stable; that of *A. canaliculatum* was projected to shift northward under the high-emission scenario; and those of *A. orientale* and *A. plantago-aquatica* exhibited low-suitability contraction with high-suitability expansion. Model projections indicate that the southern portions of their current suitable habitats may lose suitability, but whether actual populations would decline requires field validation. (4) Centroid displacement and niche conservatism analyses consistently supported this sensitivity gradient: *A. gramineum* displayed the most stable distribution and niche, *A. orientale* was the most climate-sensitive species, and *A. canaliculatum* and *A. plantago-aquatica* showed intermediate sensitivities. (5) Most species pairs showed high niche overlap, whereas the niche identity test for *A. orientale* and *A. plantago-aquatica* did not reach statistical significance. These findings suggest that priority should be given to further field surveys and conservation planning in the core suitable habitats of *A. orientale* and *A. plantago-aquatica*, and long-term monitoring should be conducted in the potential expansion areas of *A. canaliculatum*.

## Figures and Tables

**Table 1:** Performance evaluation metrics of the MaxEnt models for the four *Alisma* species under current climate conditions.

Species	Period	Training AUC	Test AUC	TSS (Mean ± SD)	Kappa (Mean ± SD)	Boyce (Mean ± SD)
*Alisma canaliculatum*	current	0.9853	0.9787	0.9463 ± 0.0235	0.8879 ± 0.0101	0.2524 ± 0.0947
*Alisma gramineum*	current	0.9910	0.9878	0.9461 ± 0.0360	0.8688 ± 0.0206	0.2846 ± 0.2141
*Alisma orientale*	current	0.9192	0.9173	0.8795 ± 0.0432	0.6680 ± 0.0219	0.3702 ± 0.1018
*Alisma plantago-aquatica*	current	0.9681	0.9648	0.8847 ± 0.0176	0.8589 ± 0.0076	0.4234 ± 0.0659

**Table 2:** Contribution rates and suitable ranges (>50% suitability) of the dominant environmental factors in the MaxEnt models for the four *Alisma* species.

Species	Variables	Contribution (%)	Suitable Range (>50% Suitability)	Unit
*Alisma* *canaliculatum*	bio11	40.7	−0.42 to 9.82	°C
	bio03	26.1	23.11 to 32.35	%
	bio09	16.7	0.87 to 11.83	°C
	bio18	11.3	425.95 to 835.75	mm
	bio15	2.1	48.99 to 84.93	%
*Alisma* *gramineum*	hf_v2geo1	33.6	37.536 to 198.186	–
	bio19	24.6	3.93 to 15.70	mm
	bio04	24.1	1056.87 to 1451.45	°C × 100
	bio09	6.9	−16.10 to −2.19	°C
	bio03	4.3	24.65 to 33.12	%
*Alisma* *orientale*	bio09	36.8	−10.79 to 10.84	°C
	bio15	26.6	≥92.87	%
	bio11	19.2	−10.78 to 10.76	°C
	bio03	10.8	≤32.14	%
	bio14	2.9	≤17.14	mm
*Alisma* *plantago-aquatica*	hf_v2geo1	37.2	37.536 to 175.236	–
	bio15	17.3	72.58 to 138.53	%
	bio18	15.5	293.12 to 947.38	mm
	bio09	15.5	−10.51 to 13.08	°C
	bio03	8.6	23.37 to 32.87	%

**Table 3:** Changes in potential suitable habitat area of the four *Alisma* species under different climate scenarios.

Species	Period	Climate Scenarios	Suitable Area	Optimal Area	Range Expansion	No Change	Range Contraction
*Alisma* *canaliculatum*	current	—	140.163	37.142	—	—	—
	2041–2060	SSP1-2.6	134.062	79.274	40.809	172.527	4.778
		SSP2-4.5	144.690	72.655	53.198	164.148	13.157
		SSP5-8.5	121.530	117.612	65.346	173.797	3.508
	2081–2100	SSP1-2.6	135.355	69.804	35.917	169.242	8.062
		SSP2-4.5	133.921	90.929	52.422	172.428	4.876
		SSP5-8.5	161.965	193.800	181.218	174.547	2.758
*Alisma* *gramineum*	current	—	162.612	16.223	—	—	—
	2041–2060	SSP1-2.6	169.256	18.328	15.331	172.253	6.582
		SSP2-4.5	151.109	18.285	9.289	160.105	18.730
		SSP5-8.5	167.508	21.934	18.287	171.156	7.680
	2081–2100	SSP1-2.6	158.625	19.410	8.833	169.201	9.634
		SSP2-4.5	159.900	20.344	14.776	165.468	13.367
		SSP5-8.5	166.025	23.792	20.492	169.326	9.510
*Alisma* *orientale*	current	—	597.284	157.465	—	—	—
	2041–2060	SSP1-2.6	589.797	199.018	70.929	717.886	36.862
		SSP2-4.5	558.941	235.688	73.013	721.617	33.132
		SSP5-8.5	574.177	240.997	108.717	706.457	48.291
	2081–2100	SSP1-2.6	562.241	239.366	86.932	714.674	40.074
		SSP2-4.5	599.561	253.411	128.180	724.792	29.956
		SSP5-8.5	510.820	303.523	131.677	682.666	72.083
*Alisma* *plantago-aquatica*	current	—	343.145	51.553	—	—	—
	2041–2060	SSP1-2.6	333.017	56.726	13.213	376.530	18.168
		SSP2-4.5	327.400	70.577	20.327	377.650	17.047
		SSP5-8.5	361.506	59.506	38.378	382.633	12.064
	2081–2100	SSP1-2.6	341.052	63.186	27.581	376.657	18.041
		SSP2-4.5	321.036	97.904	39.648	379.293	15.405
		SSP5-8.5	279.038	106.716	24.907	360.846	33.851

## Data Availability

Species occurrence records were obtained from the Global Biodiversity Information Facility (GBIF; https://www.gbif.org/, Available online: https://doi.org/10.15468/dl.2cwazv (accessed on 15 January 2026) [15]), the China Digital Herbarium (https://www.cvh.ac.cn/), and the China Plant Image Library (https://ppbc.iplant.cn/).

## Supplementary Materials

The following supporting information can be downloaded at: https://www.mdpi.com/article/10.3390/biology15181649/s1, Figure S1: Response curves of key environmental factors in the MaxEnt models for the four Alisma species; Figure S2: Locations of the main provinces and geographical features mentioned in the text, including the Sichuan Basin, Yangtze River, Inner Mongolia, Loess Plateau, and Yunnan–Guizhou Plateau; Table S1: Environmental variables and their abbreviations used in this study. A total of 19 bioclimatic variables (bio1–bio19), one topographic variable (elevation), one human activity disturbance factor, and six soil environmental factors were included.
