# Supplementary material for: Predicting Potential Suitable Habitats of Four Alisma Species in China Under Climate Change Scenarios
Source: Biology (Basel). 2026 Sep 18;15(18):1649. doi: 10.3390/biology15181649 (PMC13604508; doi:10.3390/biology15181649)
Supplement: Supplementary file 1 [file biology-15-01649-s001.zip › biology-4546700-supplementary.pdf]

Predicting Potential Suitable Habitats of four *Alisma* Species in China under Climate Change Scenarios

Yulu Li, Fu Huang, Qingqing Li, Jinghui Zhang, Shujian Zhang, Chao Peng, Xiaohong Li* and Qitao Su*

Key Laboratory of Jiangxi Province for Biological Invasion and Biosecurity, School of Life Sciences, Jinggangshan University, Ji'an 343009, China

* Correspondence: 9920060080@jgsu.edu.cn (X.L.); suqitao@jgsu.edu.cn (Q.S.)

Supp. Table S1. Environmental variables and their abbreviations used in this study. A total of 19 bioclimatic variables (bio1–bio19), one topographic variable (elevation), one human activity disturbance factor, and six soil environmental factors were included.

Variables	Abbreviations
Annual mean temperature	bio1
Mean diurnal range	bio2
Isothermality	bio3
Temperature seasonality	bio4
Max. temperature of warmest month	bio5
Min. temperature of coldest month	bio6
Temperature annual range	bio7
Mean temp of wettest quarter	bio8
Mean temp of driest quarter	bio9
Mean temp of warmest quarter	bio10
Mean temp of coldest quarter	bio11
Annual precipitation	bio12
Precipitation of wettest month	bio13
Precipitation of driest month	bio14
Precipitation seasonality	bio15
Precipitation of wettest quarter	bio16
Precipitation of driest quarter	bio17
Precipitation of warmest quarter	bio18
Precipitation of coldest quarter	bio19
Elevation	elev
human activity	ha
slope	slo
aspect	asp
Top soil texture	t_texture
Soil reference depth	ref_depth
Soil available water content	awc_class
Soil phase	phase1

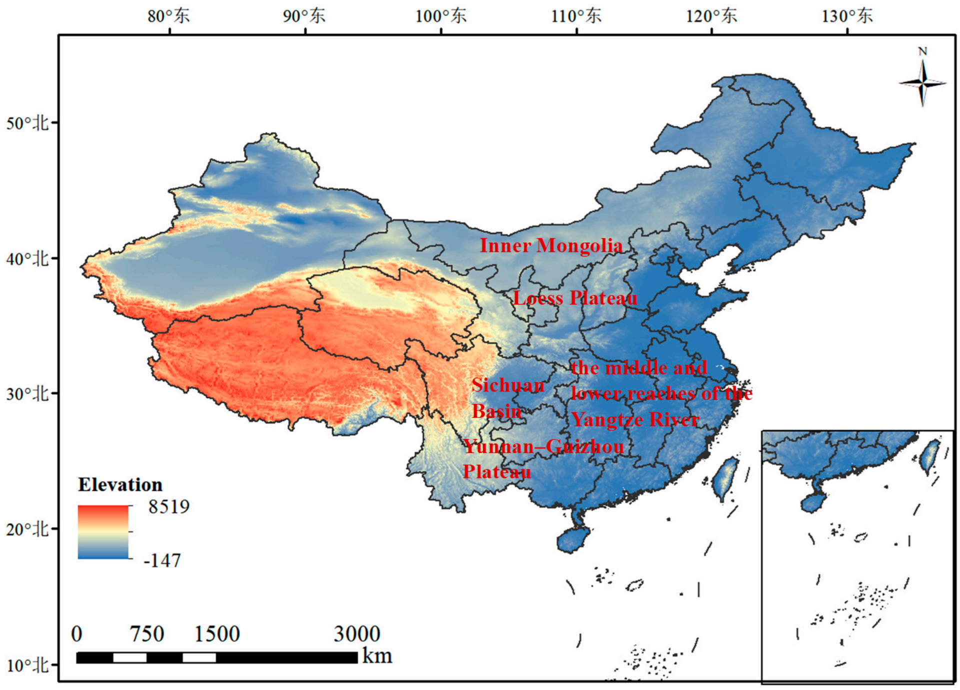

Supp. Figure S1. Locations of the main provinces and geographical features mentioned in the text, including the Sichuan Basin, Yangtze River, Inner Mongolia, Loess Plateau, and Yunnan–Guizhou Plateau.

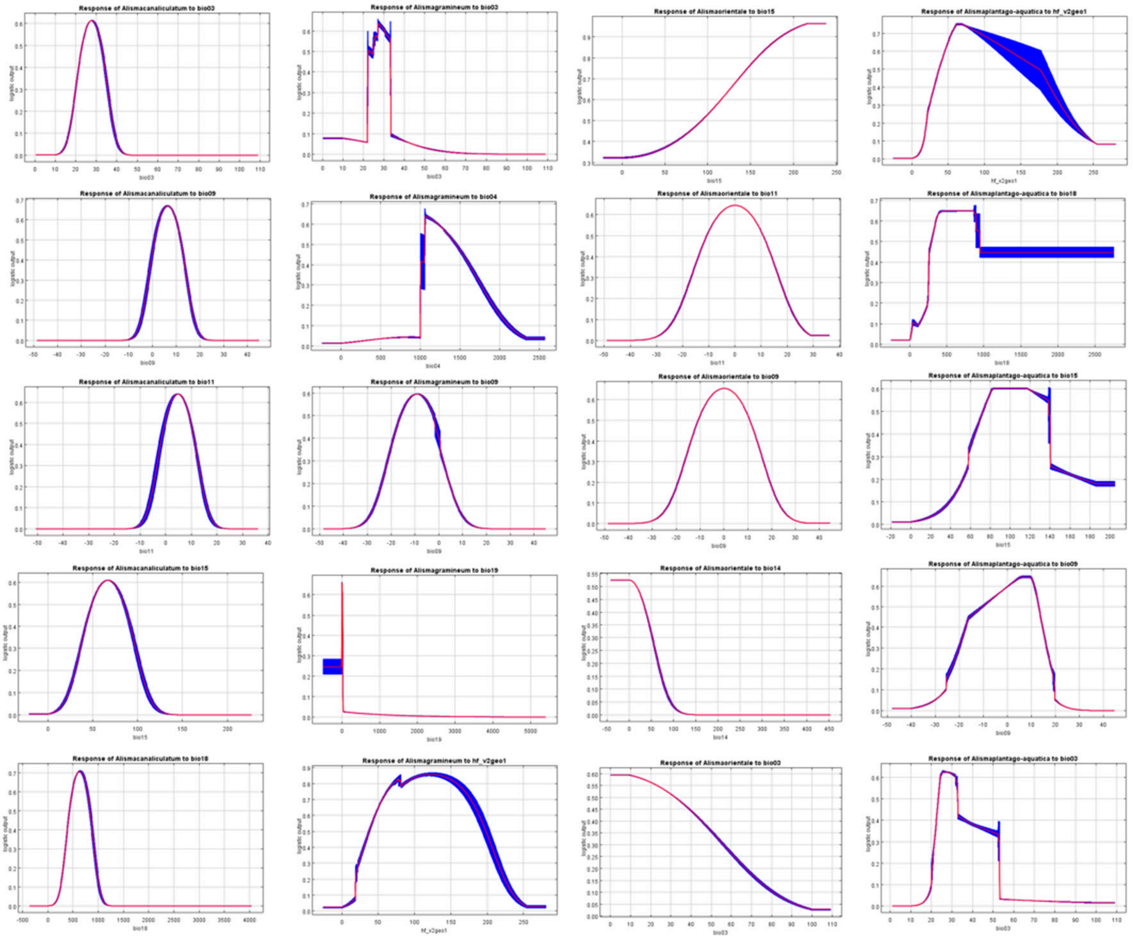

Supp. Figure S2. Response curves of key environmental factors in the MaxEnt models for the four *Alisma* species. The figure contains 20 panels, arranged by column for *A. canaliculatum* (first column), *A. gramineum* (second column), *A. orientale* (third column), and *A. plantago-aquatica* (fourth column), showing the univariate response relationships between each species and the top five dominant environmental variables contributing to the respective model. In each panel, the x-axis represents the value of the environmental variable, and the y-axis represents the logistic output probability of species presence. Red lines indicate the mean response from 10 replicate MaxEnt runs, and blue shaded areas represent the mean ± 1 standard deviation, reflecting the uncertainty of model predictions.
